# Short‐term ventriculo‐arterial coupling and myocardial work efficiency in preterm infants undergoing percutaneous patent ductus arteriosus closure

**DOI:** 10.14814/phy2.15108

**Published:** 2021-11-21

**Authors:** Adrianne R. Bischoff, Amy H. Stanford, Patrick J. McNamara

**Affiliations:** ^1^ Division of Neonatology Department of Pediatrics University of Iowa Iowa City Iowa USA; ^2^ Department of Internal Medicine University of Iowa Iowa City Iowa USA

**Keywords:** myocardial work, patent ductus arteriosus, percutaneous, transcatheter, ventriculo‐arterial coupling

## Abstract

Definitive closure of a patent ductus arteriosus (PDA) causes significant changes in loading conditions of the left ventricle (LV) which can lead to cardiorespiratory instability including hypotension, low cardiac output, oxygenation, and ventilation impairment. Physiological insights of the adaptation of the LV can be gained by looking at ventriculo‐arterial coupling (VAC) and myocardial work‐energetics. We conducted a retrospective cohort study of preterm infants with echocardiographic assessment of VAC parameters, including end‐systolic and arterial elastance (E_ES_, E_A_), and myocardial work indices derived from longitudinal strain analysis before and 1‐h after percutaneous PDA closure. A total of 35 patients were included with mean [±SD] age at intervention of 30.8 ± 9.9 days and median [IQR] weight of 1130 [995, 1318] grams. There was a reduction in preload and stroke volume, an increase in E_A_ (38.6 ± 11.4 vs. 60 ± 15.1 mmHg/ml/kg, *p* < 0.001) and in E_ES_ (72 [61.5, 109.8] vs. 91.6 [72.2, 125.2] mmHg/ml/kg, *p* = 0.003) post‐closure. Myocardial work indices reduced after PDA closure, including global work efficiency (93.9 ± 2.3 vs. 91.1 ± 3.6%, *p* < 0.001). A total of 17 (48.6%) patients developed post‐closure instability which was associated with younger age, lower preload, and higher E_A_ and E_ES_. Percutaneous PDA closure is associated with major short‐term changes in VAC and myocardium energetics, which may provide novel insights on the physiology of PDA closure and on the differential vulnerability to changes in loading conditions.

## Graphical abstract

Definitive closure of patent ductus arteriosus is associated with significant changes in ventriculo‐arterial coupling and myocardial work indices including reduced preload, stroke volume, global longitudinal strain, and ventricular efficiency, with increased arterial elastance. Patients who develop cardiorespiratory instability after the procedure have higher arterial and end‐systolic elastance. These data may provide an enhanced understanding of the physiology of patent ductus arteriosus closure and serve as a guide to optimize care and provide targeted therapies in vulnerable preterm infants.

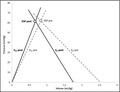

## 1 INTRODUCTION

Patent ductus arteriosus (PDA) is associated with significant morbidity, particularly in preterm infants (Weisz et al., 2017). Definitive closure, whether surgical or through a percutaneous approach, leads to a sudden change in loading conditions of the left ventricle (LV) which can be associated with significant physiological derangements and lead to hypotension, low cardiac output, oxygenation, and ventilation impairment (Giesinger et al., 2019). Several echocardiography studies have investigated the changes in LV function associated with increased afterload following PDA closure (El‐Khuffash et al., 2012; Jain et al., 2012; McNamara et al., 2010; Noori et al., 2007; Teixeira et al., 2008; Ting et al., 2016). There are, however, several limitations on the use of traditional echocardiographic markers of LV systolic performance. For instance, the estimation of ejection fraction (EF) through different methods is largely influenced by loading conditions. Structural changes that modify end‐diastolic volume (EDV) have a strong influence on EF at a given level of myocardial contractility and stroke volume (SV) (Konstam & Abboud, 2017). Therefore, although EF is a marker of LV performance, it cannot be interpreted as an isolated index of LV contractility (Bussmann & El‐Khuffash, 2019). Furthermore, M‐mode estimates of EF are angle‐dependent and based on linear geometrical assumptions that may not accurately represent true three‐dimensional volume (Cameli et al., 2016; Teichholz et al., 1976; Zacà et al., 2010). Deformation analysis with longitudinal strain using two‐dimensional speckle tracking echocardiography (2D‐STE) appears to be more sensitive than EF in detecting subtle changes in LV performance but is also load‐dependent (Bussmann & El‐Khuffash, 2019; El‐Khuffash et al., 2012). PDA closure leads to an inverse stress‐velocity relationship (McNamara et al., 2010), which is a preload‐independent, afterload adjusted method of assessing LV contractility (Colan et al., 1984) that has similar limitations related to M‐mode methodology.

Further physiological insights on LV adaptation post‐PDA closure can be gained by assessing ventriculo‐arterial coupling (VAC). Coupling refers to the influence of the load imposed by the arterial system on ventricular systolic performance (Kass & Kelly, 1992; Monge García & Santos, 2020). Study of VAC enables enhanced understanding of the interaction between both systems to better assess cardiovascular performance and cardiac energetics (Starling, 1993). This theoretical model encompasses the relationship between arterial elastance (E_A_) and end‐systolic elastance (E_ES_) (Sunagawa et al., 1983). Nagata et al. described the changes in VAC in a cohort of preterm infant undergoing surgical ligation and demonstrated that ventricular efficiency transiently deteriorated in the first 24 h after the procedure, accompanied by an increase in E_A_ and in E_A_/E_ES_ (Nagata et al., 2013). Conversely, Gray et al. identified a pre‐procedural E_A_/E_ES_ threshold as a predictor for need of postoperative vasoactive support (Gray et al., 2017). No previous studies have assessed VAC following percutaneous PDA closure.

Lastly, advanced techniques to study LV mechanics continue to emerge. Myocardial work is a relatively novel echocardiography tool that provides information regarding ventricular systolic function and contractility. The LV pressure‐strain relationship can be obtained from echocardiography‐derived longitudinal strain and the estimated LV systolic pressure via peripheral blood pressure measurement (Roemer et al., 2021). By incorporating LV load, myocardial work analysis provides an index similar to that obtained with invasive pressure‐volume loop area, which reflects myocardial oxygen consumption (Boe et al., 2019; Suga, 1990). Combined with the estimations provided by the ventriculo‐arterial coupling model, our objective was to assess myocardial work in order to better understand LV mechanics and energetics therein providing novel mechanistic insights in the care of vulnerable infants undergoing PDA closure.

## 2 METHODS

A retrospective cohort study of neonates who underwent percutaneous PDA closure at ≤2kg at the time of intervention was conducted at a quaternary referral center (The University of Iowa Stead Family Children's Hospital) from September 2019 until May 2021. Patients were excluded from analysis if the procedure was unsuccessful (defined as patient leaving the catheterization laboratory without a device placed in the PDA) or if there was another congenital heart defect with the exception of small patent foramen ovale (PFO) and/or atrial septal defect (ASD). Comprehensive targeted neonatal echocardiography (TnECHO) is performed by the Neonatal Hemodynamics team according to a standardized protocol that includes comprehensive imaging of intracardiac anatomy, outflow tract concordance and integrity, aortic arch anatomy, pulmonary vein location/flow, and transitional shunts (Bischoff et al., 2020). Medical therapy of the PDA is indicated if a modified PDA score is ≥6 or at the discretion of the attending neonatologist (Table S1) (Rios et al., 2020). Percutaneous PDA closure is recommended if medical therapy is unsuccessful (typically after 2–3 courses of acetaminophen and/or indomethacin) or contraindicated and there is ongoing evidence of a moderate‐high volume shunt (PDA score >6). The Neonatal Hemodynamics team, in conjunction with the interventional Pediatric Cardiology team, triage morphological suitability for percutaneous closure. Once the referral for definitive PDA closure has been done, TnECHO is obtained up to 24–48 h prior to percutaneous closure to confirm ductal patency, hemodynamic significance, and aortic arch sidedness. Periprocedural care is managed according to a standardized protocol. All patients who undergo definitive closure of the PDA have a comprehensive TnECHO assessment 1‐h after closure to predict which neonates are at risk for cardiorespiratory instability. A threshold left ventricular output (LVO) less than 180 ml/min/kg was used as an indication to start prophylaxis with milrinone infusion (Jain et al., 2012). For infants with borderline LVO (180–220 ml/kg/min), other indices of LV function were considered, including longitudinal strain and a prolonged isovolumic relaxation time (>60 msec).(Giesinger et al., 2019) Milrinone infusion was initiated at 0.33 mcg/kg/min (0.2 mcg/kg/min for infants <1000 g) accompanied by a normal saline bolus of 10 ml/kg during the first hour of infusion. Treatment is typically continued for the subsequent 24–48 h depending on clinical course. Patients with systemic hypertension and/or cardiorespiratory instability received dose escalation of the milrinone infusion or treatment for a longer period at the discretion of the neonatal hemodynamics team.

### 2.1 Blood pressure assessment

Blood pressure (BP) was collected by the oscillometric method in the right arm (pre‐ductal) and in one of the calves (post‐ductal) at the end of both TnECHOs (before and 1‐h after closure) with appropriately sized cuffs when the infant was calm. When available, invasive blood pressure (from an indwelling umbilical arterial catheter or peripheral arterial line) was recorded concomitantly. For the calculations used in this study, including ventriculo‐arterial coupling and myocardial work, the BP obtained from the right arm oscillometric measurement was preferentially used.

### 2.2 Targeted neonatal echocardiography

Evaluations were performed using the Vivid E90 cardiovascular ultrasound system (GE Medical Systems) with a 12‐MHz high frequency phased‐array transducer probe. Standard two‐dimensional, M‐mode, color Doppler, pulse‐wave Doppler, and continuous‐wave Doppler images were obtained. All echocardiography analyses were performed using a dedicated workstation (EchoPAC version BT10; GE Medical Systems) by a single trained investigator, who was blinded to the clinical information to minimize bias. Three consecutive cardiac cycles were evaluated and averaged for each measurement to be used in the study.

### 2.3 LV evaluation [conventional imaging]

LVO was obtained by placing a pulse‐wave Doppler perpendicular to the aortic valve in the apical five‐chamber view, with the angle of insonation parallel to the left ventricular outflow tract. The area under the wave form of the aortic systolic beat was traced to obtain the velocity time integral (VTI) and the heart rate. The annulus of the aortic valve was measured, from the parasternal long axis, between hinge points with the valve open at the end of ejection. LVO (expressed in ml/min/kg) was calculated by multiplying the aortic cross sectional area [calculated as: (aortic radius^2^ π)] by VTI and heart rate and indexed to weight (kg) (El‐Khuffash & McNamara, 2011; Ficial et al., 2013). Stroke volume was obtained by indexing LVO to heart rate obtained in the same view. The Simpson's biplane method was used to estimate end‐diastolic and end‐systolic volumes (EDV and ESV, respectively) by tracing the endocardial border in the apical four‐ and two‐ chamber views of the LV in end‐diastole and end‐systole, respectively. Stroke volume of the Simpson's biplane method was derived from ESV and EDV. All volumes obtained from the Simpson's biplane method were indexed to weight.

### 2.4 Ventriculo‐arterial coupling

**FIGURE 1:**
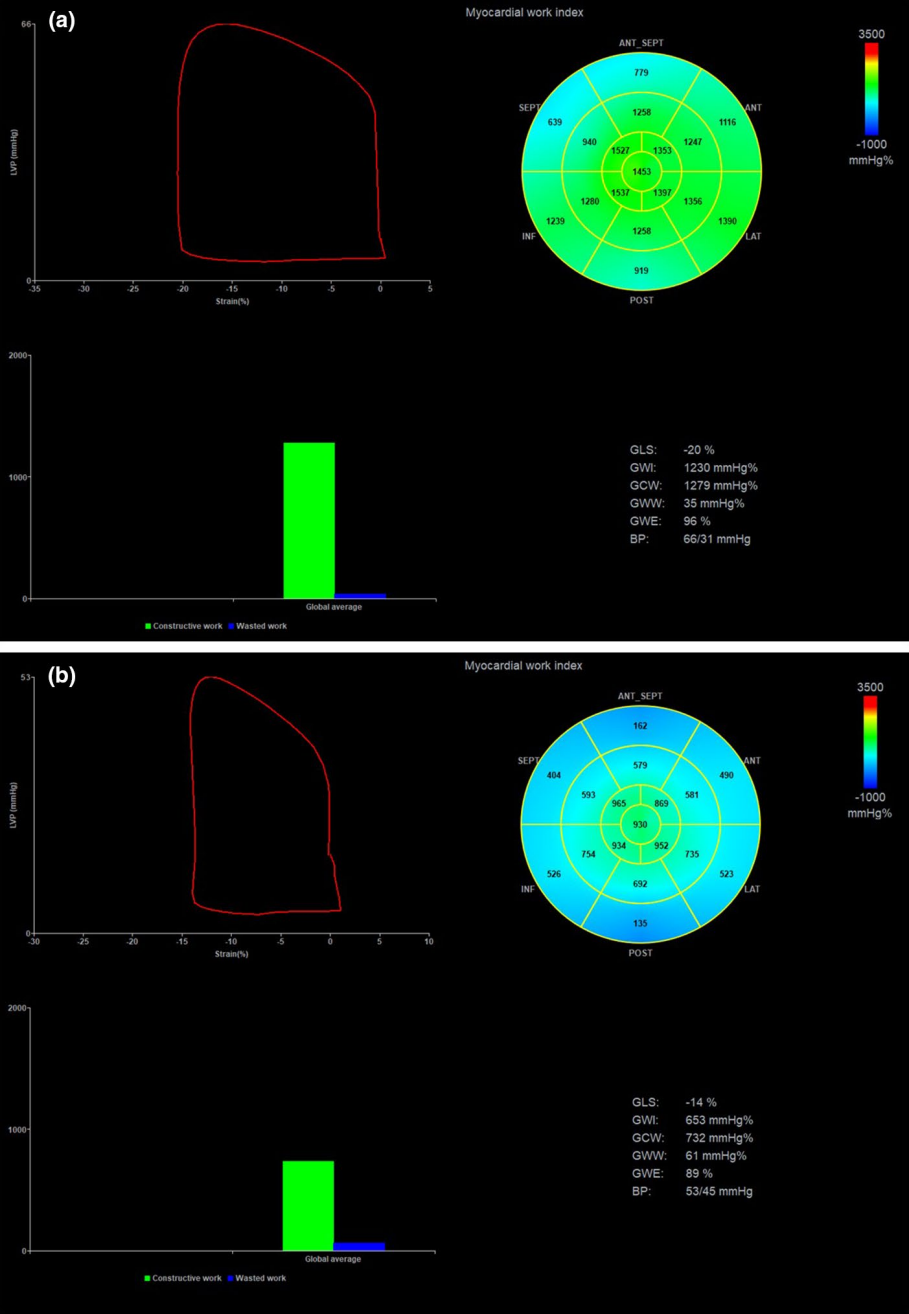
Pressure‐strain loop curve and myocardial work indices in a patient prior (panel a) and 1‐h after (panel b) percutaneous closure of patent ductus arteriosus

To assess VAC, mathematical formulas were used to estimate E_A_ and E_ES_. E_A_ encompasses both mean and pulsatile loading on the heart and represents the negative slope from the pressure‐volume loop that joins the end‐diastolic volume and end‐systolic pressure points (Borlaug & Kass, 2008; Sunagawa et al., 1983). Conversely, E_ES_ is determined from the slope of the end‐systolic pressure‐volume relationship and is considered a relatively load‐independent index of LV contractility (Figure 1) (Sagawa et al., 1977). While baseline E_ES_ describes the intrinsic characteristics of the myocardium that are modulated by geometry, structure, and functional properties, acute changes in LV contractility can change the slope of the curve (Kass, 2002). The graphic representation of stroke work (SW) is the area under the systolic segment of the pressure‐volume loop curve, expressing the work performed to propel blood from the ventricle to the aorta (Burkhoff & Sagawa, 1986; Nozawa et al., 1988). In turn, the pressure‐volume loop area (PVA) is circumscribed by the end‐systolic and end‐diastolic pressure‐volume loop relationships and the systolic segment of the pressure‐volume loop curve (Figure S1, panel a) (Burkhoff & Sagawa, 1986). It therefore represents the sum of SW and the end‐systolic potential energy and it is linearly correlated with myocardial oxygen consumption (Suga, 1990; Suga et al., 1981). The ratio between SW/PVA represents the energetic efficiency of the LV (Suga et al., 1981; Sunagawa et al., 1985).

All the VAC parameters were obtained by estimating end‐systolic pressure (ESP) and using ventricular volumes obtained from the TnECHOS. We used different methods to calculate ESP as per the following formulas: ESP_1_ = SBP × 0.9 (Baumgartner et al., 2018; Chowdhury et al., 2016; Kelly et al., 1992), ESP_2_ = [(2 × SBP) + DBP]/3 (Engel et al., 2014; Kelly et al., 1992; Kuznetsova et al., 2012), and ESP_3_ = [(SBP × 0.4) + (DBP × 0.6) + 1.4] (Iwahara et al., 1991), where SBP represents the systolic BP and DBP represents the diastolic BP. The first method (ESP_1_) has been described more often in the literature and therefore was used as the primary outcome in this study. To estimate E_A,_ we used two different methods to estimate stroke volume obtained by echocardiography methods. E_A_ calculations have been previously described using M‐mode measurements (Baumgartner et al., 2018; Engel et al., 2014). However, due to the geometrical assumptions that are required to estimate volumes using M‐mode and its specific limitations in neonates, we chose two alternate methods that have higher reliability (Cameli et al., 2016; Zacà et al., 2010). The first was based on SV, estimated from the Simpson's biplane method, while the second estimated SV by tracing the LVO as previously described. E_A_ was estimated by diving ESP over SV as per the following (Kelly et al., 1992): E_A1_ = ESP_1_ / SV by Biplane, E_A2_ = ESP_1_/SV by LVO, E_A3_ = ESP_2_/SV by Biplane, E_A4_ = ESP_2_/SV by LVO, E_A5_ = ESP_3_/SV by Biplane, and E_A6_ = ESP_1_/SV by LVO. For estimation of E_ES_, end‐systolic volume (ESV) was obtained from the Simpson's biplane method and all formulas of ESP were used as follows: E_ES1_ = ESP_1_/ESV, E_ES2_ = ESP_2_/ESV, E_ES3_ = ESP_3_/ESV (Chantler & Lakatta, 2012). The estimations of VAC (E_A_/E_ES_) were performed using both methods of SV estimation: VAC_1_ = ESV/SV by Biplane method; VAC_2_ = ESV/SV by LVO method (Engel et al., 2014; Gray et al., 2017; Nagata et al., 2013). Finally, ventricular efficiency (VE) was derived from the VAC formulas: VE_1_ = 1/1 + VAC_1_ * 0.5, VE_2_ = 1/1 + VAC_2_ * 0.5 (Nagata et al., 2013; Nozawa et al., 1988). Of note, SV estimated by Biplane and blood Doppler are not comparable (Phad et al., 2018); therefore, for consistency purposes we opted to use the formulas derived from the Biplane method and ESP_1_ (E_A1_, E_ES1_, VAC_1_, and VE_1_) as the primary outcomes.

Ventriculo‐arterial coupling graphs prior to and after the procedure were drawn by using the volumes estimated from the Simpson's biplane method and the ESP_1_. The graph represents the mean values for E_A1_, E_ES1_ and the ESP_1_ volume point (ESP_1_/ESV). The intercept with the x‐axis represents V_0_ for the E_ES1_ line and EDV for the E_A1_ line.

### 2.5 Myocardial work

Automated functional imaging (AFI) (GE, Milwaukee, WI) using 2D‐STE was performed from the apical four‐, two‐ and three‐chamber views of the LV (Tran et al., 2016). A frame rate of 80–100 frames/sec was used for storage and analysis but only images that were optimized to visualize the myocardial walls were used. LV contouring was drawn at the endocardial border from mitral annulus on one side to mitral annulus on the opposite side. The smallest width of region of interest (ROI) available by the software was used for tracing. Attempts were made to avoid tracing of the pericardium which could underestimate myocardial work (Smiseth et al., 2021). Tracking was automatic and its acceptability was visually inspected, irrespective of the software's automatic suggestion, and the appropriate boundaries confirmed. To generate myocardial work data, satisfactory tracking of all myocardial segments from all three different views is required. If the tracking was deemed to be suboptimal, the endocardial border was retraced; however, if satisfactory tracking was not achieved within 5 minutes, data were excluded from analysis. Valvular event times were set by two‐dimensional evaluation of the apical three‐chamber view. The period of interest was defined from mitral valve closure to mitral valve opening. LV pressure‐strain loops and bull's eye plots of longitudinal strain, myocardial work, and myocardial efficiency were generated (Figure 1). Global values were collected for the following variables: longitudinal strain, work index, constructive work, wasted work, and work efficiency. Global work index (GWI) represents the total work performed by the LV during mechanical systole, isovolumic contraction, and relaxation (Roemer et al., 2021). Constructive work represents the work during LV systole that is synchronous with the timing of the cardiac cycle, including shortening of the muscle during systole and lengthening of the muscle during the isovolumic relaxation time (Manganaro et al., 2019). On the contrary, wasted work occurs when contraction or lengthening occur at the wrong time of the cardiac cycle and adds a metabolic burden to the LV (Manganaro et al., 2019). Finally, global work efficiency (GWE) represents the ratio of constructive work over the sum of constructive and wasted work and reflects the efficiency of the energy expended throughout the cardiac cycle (Boe et al., 2019; Manganaro et al., 2019; Roemer et al., 2021; Smiseth et al., 2021).

### 2.6 Outcomes

The primary outcome was change in VAC parameters and myocardial work after percutaneous PDA closure. The secondary outcome was post‐closure cardiorespiratory instability defined as either: ventilation and/or oxygenation failure and/or systolic hypotension and/or escalation in cardiovascular support in the first 24 h after the procedure. Oxygenation failure was defined as an absolute increase of at least 20% in fraction of inspired oxygen (FiO_2_) or mean airway pressure (MAP) compared to pre‐closure value, for at least 1‐h, in the first 24 h of post‐closure. Ventilation failure was defined as need for rescue high frequency ventilation in patients who were on conventional mechanical ventilation because of inability of conventional settings to maintain adequate ventilation support in the first 24 h of post‐closure. Systolic hypotension was defined as systolic arterial pressure below the 3^rd^ percentile for age for at least 1‐h in the first 24 h of the procedure. Escalation in cardiovascular support was defined as an increase in inotrope score by >20% of the pre‐closure value or need to initiate a new agent (not including milrinone) in response to hypotension, shock and/or LV systolic dysfunction on echocardiography (Jain et al., 2012; Ting et al., 2016).

### 2.7 Statistical analysis

Descriptive statistics were used for demographics and clinical data. The Shapiro–Wilk test was used to test continuous variables for normality. Mean with standard deviation and median with interquartile range were calculated for data with normal and non‐normal distribution, respectively. Pre‐ and post‐procedural variables were compared using paired *t*‐test for normally distributed variables and Wilcoxon Signed‐Rank test for non‐normally distributed variables with symmetry. Sign test was used for non‐normally distributed variables without symmetry. The net change in various ventriculo‐arterial coupling, myocardial work indices, and echocardiography measurements was calculated. Patients with cardiorespiratory instability were compared to those without instability using parametric (Student *t*‐test) and nonparametric tests (Mann–Whitney) as appropriate for continuous variables. Categorical variables were presented as frequencies (%) and compared using the Chi‐squared test or Fisher's exact test. Intra‐ and inter‐observer reliability testing were performed for myocardial work analysis. Testing was performed for a single set of defined images (analysis‐reanalysis) in 10 randomly selected TnECHOs (5 prior and 5 after PDA percutaneous closure). Intra‐observer reliability was assessed by one investigator (A.B.) who performed offline analysis of the same TnECHO studies 4 weeks apart to reduce recall bias, while inter‐observer was assessed by a second investigator (A.S.) who was unaware of the previous results. Agreement between investigators was evaluated using the Bland–Altman method to calculate the bias (mean difference) and the 95% limits of agreement 1.96 SD around the mean difference).(Bland & Altman, 1986) Results were considered significant if *p* < 0.05, and all results where *p* < 0.1 were reported numerically as nonsignificant trends. There are no previous studies assessing changes in myocardial work in preterm infants undergoing PDA closure, therefore sample size calculation was not possible. We used a convenience sample size by including all patients undergoing the procedure during the study period. Data were analyzed using SPSS version 27 statistical software (IBM).

## 3 RESULTS

During the study period, 41 neonates were referred for percutaneous PDA closure. A total of six patients were excluded from analysis: 4 patients were >2kg at the time of the procedure (considered low‐risk for heart dysfunction); 1 patient had procedure cancellation as the PDA was considered too small for definitive closure upon arrival to the catheterization laboratory; 1 patient had incomplete clinical and echocardiographic data. Ultimately, a total of 35 neonates (71% males) were included in the final cohort whose mean [±SD] birth weight was 729 ± 197 grams and gestational age was 25.07 ± 1.71 weeks. Mean age at intervention was 30.8 ± 9.9 days and median [IQR] weight and postmenstrual age were 1130 [995, 1318] grams and 29.1 [28.2, 30.9] weeks, respectively. All patients were mechanically ventilated prior to the procedure, of whom 28 (80%) were managed with high frequency jet ventilation and the remainder were supported by conventional mechanical ventilation (pressure‐control synchronized intermittent mandatory ventilation). Baseline respiratory illness severity was a mean [±SD] MAP of 12.1 ± 2.2 cmH_2_O, median [IQR] FiO_2_ of 35% [31, 50], and respiratory severity score (calculated as MAP*FiO_2_ (Bischoff et al., 2021)) of 4.48 [3.5, 6.48]. None of the patients were receiving cardiovascular medications prior to the procedure or at the 1‐h post‐closure TnECHO. Table 1 depicts the post‐procedural clinical outcomes.

**TABLE 1:** Clinical outcomes of preterm infants undergoing percutaneous patent ductus arteriosus closure

Characteristics: (*n* = 35)	
Received milrinone prophylaxis	24 (68.6)
Oxygenation failure	15 (42.9)
Ventilation failure	3 (8.6)
Composite of oxygenation and/or ventilation failure	16 (45.7)
Systolic hypotension	1 (2.9)
Escalation in cardiovascular support post‐procedure	4 (11.4)
Composite of systolic hypotension and/or cardiovascular medications	4 (11.4)
Composite of respiratory oxygenation and/or ventilation failure and/or systolic hypotension and/or escalation in cardiovascular support post‐procedure	17 (48.6)

Note

Results presented in frequency (%).

Abbreviation: PDA, patent ductus arteriosus.

### 3.1 Ventriculo‐arterial coupling and myocardial work

**FIGURE 2:**
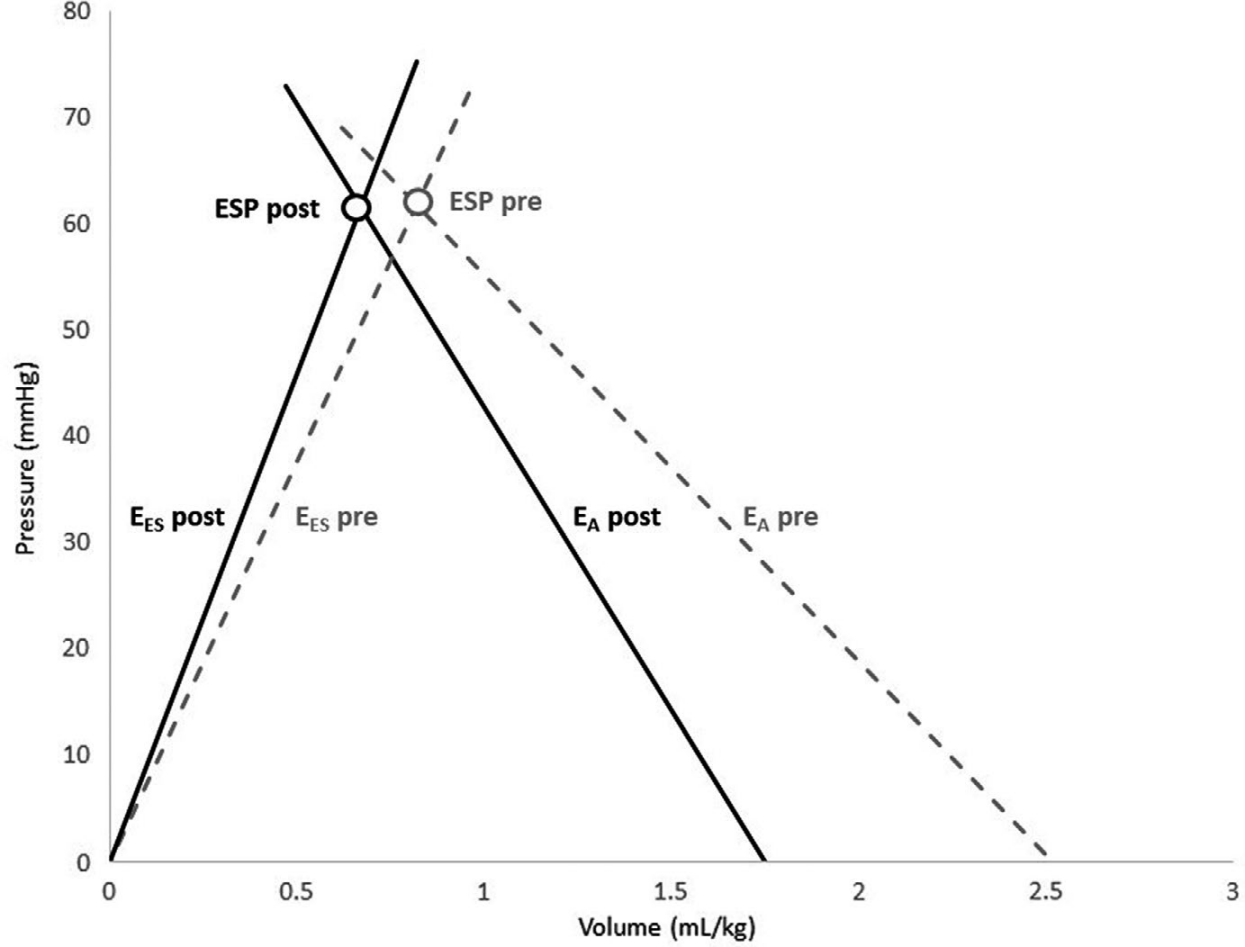
Comparison of the corresponding ventricular‐arterial coupling (E_A_/E_ES_) prior (gray dotted line) and 1‐h after (black line) percutaneous closure of patent ductus arteriosus. The slope of E_A_ and E_ES_ is steeper after PDA closure

Table 2 and Table S2 depict the changes in blood pressure, VAC parameters, and myocardial work indices in neonates undergoing percutaneous PDA closure. There was a notable decrease in preload and SV after the procedure. Arterial elastance and end‐systolic elastance increased after the procedure. Figure 2 depicts the changes in ventriculo‐arterial coupling before and after percutaneous PDA closure. Preload is reduced and the slopes of E_A_ and E_ES_ are steeper after PDA closure, with a smaller area under the curve. Decreases in ventricular efficiency were noticed after the procedure as estimated by the VAC formulas as well as by the pressure‐strain loops of myocardial work (Table 2). Figure 1 depicts an example of the pressure‐strain loop curves and the bull's eye map generated by the software to calculate indices of myocardial work in a patient prior (panel a) and after (panel b) percutaneous PDA closure. The area of the pressure‐strain loop is reflective of a decrease in myocardial work indices after PDA closure.

**TABLE 2:** Pre‐ and post‐procedural oscillometric blood pressure, ventriculo‐arterial coupling, and myocardial work indices in preterm infants undergoing percutaneous closure of patent ductus arteriosus (*n* = 35)

	Pre‐closure	1 h Post‐closure	*p*
Systolic BP (mmHg)	68.7 ± 8.8	68.3 ± 12.1	NS
Diastolic BP (mmHg)	34.6 ± 10.8	35.5 ± 8.1	NS
ESP_1_ (mmHg)	61.8 ± 7.9	61.5 ± 10.9	NS
Left ventricular output (ml/min/kg)	304 ± 79	160 ± 44	0.03
Ejection fraction by Biplane (%)	68.12 ± 6.53	61.96 ± 7.92	<0.001
Stroke volume by Biplane (ml/kg)	1.7 ± 0.41	1.07 ± 0.26	<0.001
Stroke volume by LVO (ml/kg)	1.83 ± 0.48	1.05 ± 0.29	<0.001
End‐systolic volume Biplane (ml/kg)	0.82 ± 0.29	0.67 ± 0.24	<0.001
End‐diastolic volume Biplane (ml/kg)	2.52 ± 0.63	1.74 ± 0.42	<0.001
E_A1_ (mmHg/ml/kg)	38.6 ± 11.4	60 ± 15.1	<0.001
E_A2_ (mmHg/ml/kg)	38.4 ± 12.6	60.2 ± 19.3	<0.001
E_ES1_ (mmHg/ml/kg)	72 [61.5, 109.8]	91.6 [72.2, 125.2]	0.003
VAC_1_	0.48 ± 0.14	0.64 ± 0.22	<0.001
VE_1_	0.8 ± 0.04	0.76 ± 0.06	<0.001
GLS (%)	−21.07 ± 2.64	−15.48 ± 2.83	<0.001
GWI (mmHg%)	1252 ± 217	923 ± 41	<0.001
GCW (mmHg%)	1298 ± 219	970 ± 223	<0.001
GWW (mmHg%)	70 ± 32	79 ± 37	NS
GWE (%)	93.9 ± 2.3	91.1 ± 3.6	<0.001

Note

Results presented in mean ± SD, median [IQR]. Paired *t*‐test was used for normally distributed variables and Wilcoxon Signed‐Rank test for non‐normally distributed variables with symmetry. Sign test was used for non‐normally distributed variables without symmetry.

Abbreviations: BP, blood pressure; E_A_, arterial elastance; E_ES_, end‐systolic elastance; ESP, end‐systolic pressure; ESV, end‐systolic volume; GCW, global constructive work; GLS, global longitudinal strain; GWE, global work efficiency; GWI, global myocardial work index; GWW, global wasted work; LVO, left ventricular output; SV, stroke volume; VAC, ventriculo‐arterial coupling; VE, ventricular efficiency.

### 3.2 Association with post‐closure cardiorespiratory instability

A total of 17 (48.5%) patients developed post‐PDA closure instability which was predominantly due to oxygenation and/or ventilation failure (Table 1). Patients with instability were younger, had lower pre‐procedural preload, higher pre‐ and post‐procedural E_A_, and there was a nonstatistically significant trend toward higher pre‐ and post‐procedural E_ES_ (Table 3, Table S3). The net change in stroke volume was less pronounced in patients who developed instability [−0.58 ± 0.3 vs. −0.68 ± 0.5 ml/kg estimated by Simpson's Biplane (*p* =< 0.001), −0.65 ± 0.4 vs. −0.89 ± 0.5 ml/kg estimated by LVO (nonsignificant)]. Changes in global longitudinal strain and in myocardial work indices were more pronounced in the infants who developed instability, although these did not reach statistical significance; specifically, GLS decreased by 6.7 ± 3 versus 4.3 ± 3.3% while GWE decreased by 3.5 ± 4.4 versus 2 ± 3.3% in infant with and without instability, respectively.

**TABLE 3:** Clinical and targeted neonatal echocardiography characteristics of patients with post‐patent ductus arteriosus closure respiratory and/or cardiovascular instability versus patients who remained clinically stable

	With cardiorespiratory instability (*n* = 17)	Without cardiorespiratory instability (*n* = 18)	*p*
Birth weight (grams)	674 ± 197	780 ± 188	NS
Gestational age (weeks)	24.4 ± 1.5	25.7 ± 1.6	NS
Age at intervention (days)	29.7 ± 7.6	31.9 ± 11.7	0.05
Weight at intervention (grams)	1130 ± 271	1287 ± 338	NS
Postmenstrual age (weeks)	28.3 [27.6, 30]	30 [28.9, 31]	0.01
**Pre‐procedure TnECHO**			
Systolic BP (mmHg)	68.7 ± 9.1	68.6 ± 8.8	NS
Diastolic BP (mmHg)	36.4 ± 8.3	32.9 ± 12.7	NS
ESP_1_ (mmHg)	61.9 ± 8.1	61.7 ± 7.9	NS
Left ventricular output (ml/kg/min)	293 ± 62.7	314 ± 93.8	NS
Ejection fraction by Biplane (%)	69.57 ± 7.01	66.75 ± 5.91	NS
Stroke volume by Biplane (ml/kg)	1.57 ± 0.26	1.82 ± 0.5	0.006
End‐systolic volume Biplane (ml/kg)	0.75 ± 0.24	0.88 ± 0.33	NS
End‐diastolic volume Biplane (ml/kg)	2.33 ± 0.41	2.69 ± 0.76	0.009
E_A1_ (mmHg/ml/kg)	39.4 [35.8, 43.3]	31.9 [27.3, 50.3]	0.083
E_ES1_ (mmHg/ml/kg)	74.8 [70, 113.7]	63.5 [59.8, 103.5]	NS
VAC_1_	0.48 ± 0.15	0.48 ± 0.13	NS
VE_1_	0.8 ± 0.04	0.8 ± 0.04	NS
GLS (%)	−21.8 ± 2.6	−20.4 ± 2.5	NS
GWI (mmHg%)	1288 ± 182	1207 ± 245	NS
GCW (mmHg%)	1339± 184	1248 ± 243	NS
GWW (mmHg%)	69.2 ± 32.7	71 ± 32	NS
GWE (%)	94.2 ± 2.3	93.7 ± 2.2	NS
**Post‐procedure TnECHO**			
Systolic BP (mmHg)	69 [63.5, 73.5]	65 [55.2, 76.5]	NS
Diastolic BP (mmHg)	35 [31, 40.5]	33.5 [27.7, 41]	NS
ESP_1_ (mmHg)	62.1 [57.1, 66.1]	58.5 [49.7, 68.8]	NS
Left ventricular output (ml/kg/min)	160 ± 39.2	161 ± 50.5	NS
Ejection fraction by Biplane (%)	61.94 ± 7.39	61.98 ± 8.61	NS
Stroke volume by Biplane (ml/kg)	0.99 ± 0.2	1.14 ± 0.29	NS
End‐systolic volume Biplane (ml/kg)	0.57 [0.49, 0.65]	0.68 [0.54, 0.85]	0.062
End‐diastolic volume Biplane (ml/kg)	1.6 ± 0.32	1.88 ± 0.46	NS
E_A1_ (mmHg/ml/kg)	66.1 [52.9, 77.5]	49.8 [45.5, 72.1]	0.022
E_ES1_ (mmHg/ml/kg)	111.7 [88.2, 125.8]	87.7 [58.6, 109.9]	0.083
VAC_1_	0.62 ± 0.17	0.66 ± 0.25	0.048
VE_1_	0.76 ± 0.05	0.76 ± 0.07	0.089
GLS (%)	−15 ± 2.7	−14.5 ± 3.8	NS
GWI (mmHg%)	907 ± 233	829 ± 271	NS
GCW (mmHg%)	949 ± 240	889 ± 267	NS
GWW (mmHg%)	79.4 ± 35.9	81.3 ± 42.7	NS
GWE (%)	90.7 ± 4.2	89.9 ± 5.2	NS

Note

Results presented in mean ± SD, median [IQR]. Parametric (Student *t*‐test) and nonparametric tests (Mann–Whitney) were used as appropriate for continuous variables.

Abbreviations: BP, blood pressure; E_A_, arterial elastance; E_ES_, end‐systolic elastance; ESP, end‐systolic pressure; ESV, end‐systolic volume; GCW, global constructive work; GLS, global longitudinal strain; GWE, global work efficiency; GWI, global myocardial work index; GWW, global wasted work; SV, stroke volume; VAC, ventriculo‐arterial coupling; VE, ventricular efficiency.

### 3.3 Feasibility and reliability of myocardial work analysis

Myocardial work analysis was successfully performed in 84.2% (59/70) of the echocardiography studies [80% (28/35) of the pre‐procedural and 88.5% (31/35) of the post‐procedural scans]. The main reason for inability to perform myocardial work analysis was poor tracking of the LV wall, particularly from the LV three‐ and/or two‐chamber views. Intra‐ and inter‐observer reliability testing, including the percentage bias, 95% limits of agreements and intraclass correlation coefficient are summarized in Table 4.

**TABLE 4:** Reliability of myocardial work parameters

Variable	Intra‐observer			Inter‐observer		
	Mean ± SD	Percent bias (95% LOA)	ICC (95% CI, *p* value)	Mean ± SD	Percent bias (95% LOA)	ICC (95% CI, *p* value)
GLS (%)	−20.3 ± 3.7	−1.1 (−3.04 to 0.84)	0.96 (0.57 to 0.99, <0.001)	−19.5 ± 3.8	0.6 (−1.69 to 1.17)	0.97 (0.89 to 0.99, <0.001)
GWI (mmHg%)	1158 ± 232	38.1 (−85.5 to 161.7)	0.97 (0.89 to 0.99, <0.001)	1122 ± 238	−35.1 (−187.7 to 117.5)	0.97 (0.88 to 0.99, <0.001)
GCW (mmHg%)	1188 ± 217	29.9 (−81.8 to 141.6)	0.98 (0.91 to 0.99, <0.001)	1141 ± 247	−65.3 (−342.1 to 211.8)	0.90 (0.64 to 0.97, <0.001)
GWW (mmHg%)	63.7 ± 21.5	2 (−16.8 to 20.8)	0.95 (0.81 to 0.98, <0.001)	63.7 ± 21.9	2 (−24.5 to 28.5)	0.91 (0.63 to 0.97, <0.001)
GWE (%)	93.7 ± 2	0.1 (−2 to 2.2)	0.93 (0.73 to 0.98, <0.001)	93.5 ± 2.2	−0.3 (−3.1 to 2.4)	0.90 (0.61 to 0.97, <0.001)

Note

Bland–Altman method was used to calculate the bias (mean difference) and the 95% limits of agreement 1.96 SD around the mean difference.

Abbreviations: CI, confidence interval; GCW, global constructive work; GLS, global longitudinal strain; GWE, global work efficiency; GWI, global myocardial work index; GWW, global wasted work; ICC, intraclass correlation coefficient; LOA, limit of agreement.

## 4 DISCUSSION

Percutaneous PDA closure is associated with increased arterial elastance, end‐systolic elastance, and changes in ventriculo‐arterial coupling and ventricular efficiency in the short‐term. These changes have a similar trend to what has been reported by Nagata et al. in echocardiograms performed in the first 24 h after PDA surgical ligation (Nagata et al., 2013). Following PDA closure, diastolic BP typically increases due to loss of the low resistance pulmonary vascular bed circuit and resultant increase in resting pressure against the systemic vessel walls (Giesinger et al., 2019). A variety of factors can influence BP parameters––and consequently the estimation of ESP that are used in VAC calculations––in the first 24 h after closure. These include weaning off sedatives from anesthesia, use of inotropes, afterload‐reducing agents or vasopressors, reduced cardiac output in the presence of LV systolic dysfunction, and adrenal dysfunction, etc (Giesinger et al., 2019). Unlike the previous study, our post‐procedural TnECHOs were performed at a defined timepoint, 1‐h after PDA closure, and none of the patients were receiving any cardiovascular medications at the time of assessment. This homogeneous approach creates a natural physiologic experiment which allows for the assessment of the immediate changes in coupling without other confounding factors. While we did not observe significant increases in diastolic BP nor in ESP, calculated by three different formulas, the decrease in SV with relatively maintained BP after PDA closure resulted in significant increases in E_A_.

We also observed a significant decrease in SV and EF, with increases in E_ES_, which is a load‐independent measurement that reflects intrinsic myocardium properties/contractility. This also resulted in a higher E_A_/E_ES_ with lower ventricular efficiency. When there is appropriate coupling, the transfer of blood from the heart to the periphery occurs without excessive changes in pressure, with adequate SW and energetic efficiency (Borlaug & Kass, 2008). The changes noted in the pressure‐volume loop and pressure‐strain graphs (Figures 1, 2 and S1) reflect a decrease in overall SW, with a concomitant decrease in ventricular efficiency which is related primarily to the changes in loading conditions rather than in intrinsic contractility (Nozawa et al., 1988). These findings are further validated by echocardiography myocardial work analysis that showed decreases in GWI and GWE after PDA closure. While it is not possible to perform direct comparisons between ventricular efficiency derived from mathematical equations of the VAC model and GWE, there was a decrease in both values (VE_1_ 0.8 to 0.76 and GWE from 93.9% to 91.1%) after PDA closure.

The coupling between the arterial and the ventricular systems is complex. In adults, maximal energetic efficiency occurs at a VAC around 0.5, maximal stroke work occurs when the ratio is close to 1, and an optimal coupling ratio at rest is between 0.7 and 1 (Chantler & Lakatta, 2012; Prsa et al., 2014). Children have proportionally higher E_ES_ and lower VAC than adults such that the normative data for term infants is close to 0.5 (Baumgartner et al., 2018). To our knowledge there are two previous studies describing arterial elastance, end‐systolic elastance, and ventriculo‐arterial coupling in preterm infants (Baumgartner et al., 2018; Waal et al., 2017). Baumgartner et al. described a gradual decrease in VAC over the first few weeks of life, suggestive of an adaptation from a high stroke work state in early transition to a more energy‐efficient pump later (Baumgartner et al., 2018). VAC was comparatively lower in patients with hemodynamically significant PDA shunts versus patients with no PDA throughout the transitional period up to 10 weeks of life (Baumgartner et al., 2018; Waal et al., 2017). The definition of a normal state in neonates, as well as optimal coupling ratio, is not known. One previous study described myocardial depression as an E_A_/E_ES_ ≥ 1 (Nagata et al., 2013) while Gray et al. identified a pre‐closure threshold of E_A_/E_ES_ > 0.78 as a predictor for a need of vasoactive medications after PDA ligation (Gray et al., 2017). Data on optimal level of ventriculo‐arterial coupling is limited in preterm neonates, and in particular those with shunts, but it is likely to be developmentally regulated and influenced by disease state. Further studies are needed to characterize myocardial energetics, optimal coupling, and clinical correlates in preterm infants and in different disease states.

The arterial load imposed on the LV encompasses peripheral vascular resistance, total arterial compliance, characteristic impedance as well as systolic and diastolic time intervals (Borlaug & Kass, 2008). E_A_ is determined by heart rate as well as by resistive (systemic vascular resistance) and stiffness (arterial compliance) components (Cohen‐Solal et al., 1994). Since the noninvasive calculations of E_A_ rely on peripheral BP measurements, the impact of extra‐cardiac shunts on these estimates is hard to assess. Given the different methods used to estimate BP in neonates, we elected to use the right arm oscillometric method for our ESP calculations. Noninvasive measurements are less reliable than invasive BP estimates; however, we chose the right arm for consistency in the assessment of changes before and after the procedure. Many of our patients had invasive BP available only after the procedure and these were in different locations (i.e.,: pre‐ and post‐ductal). In the presence of a significant left‐to‐right PDA shunt, the pre‐ductal circulation (including the right arm) is exposed to a higher SV; therefore, a discordance in BP parameters between upper and lower limbs is possible. Additionally, exposure of the LV to both the systemic and the pulmonary vascular beds with an open PDA is likely to impact the non‐invasive methodology to estimate E_A_. This represents a limitation in the interpretation of our results and poses questions about the most accurate noninvasive method to estimate arterial elastance in the presence of a significant transductal shunt. These changes are likely to be less relevant in the post‐procedural estimations as both pre‐ and post‐ductal circulations are exposed to the same circulating cardiac output; therefore, either upper or lower limb BP parameters are more equally reflective of the arterial load imposed to the LV.

Both active and passive cardiac muscle properties determine E_ES_, which partly explains higher E_ES_ in aging adult patients with heart failure with preserved ejection fraction (Borlaug & Kass, 2008). Similarly, preterm infants are known to have an immature pattern of diastolic performance due to underdeveloped sarcoplasmic reticulum, differences in calcium channel pumps and a disorganized myocardium fiber structure with decreased number of contractile elements (Hirose et al., 2015). This is relevant in the study of VAC as different slopes of the E_ES_ curve have a differential impact on how changes in the loading conditions affect SV, SW, and BP (Figure S1, panels b‐e). A stiffer ventricle has a much larger change in BP (and ESP) with proportionally less change in SV for different levels of EDV. Similarly, isolated increases in afterload produces a much more dramatic increase in BP (and ESP) in a stiff system, with less pronounced changes in SV but a greater increase in SW, and therefore require higher myocardial oxygen demand to achieve the same net volume transfer (Borlaug & Kass, 2008). In preterm infants, the baseline E_ES_ is relatively high, and reflects the intrinsic diastolic properties of a relatively stiff heart. After PDA closure, the significant increase in E_A_ would be expected to generate pronounced increases in BP. The magnitude of these changes is offset by the dramatic drop in preload (EDV), such that the BP remains relatively unchanged in the face of a decrease in SV. This is particularly important given that clinicians at the bedside may fail to recognize that significant physiological changes in coupling, myocardial work, and LV performance can occur in the face of relative stability of vital signs immediately after PDA closure. These changes are likely to have a profound impact on the risk of developing post‐closure cardiorespiratory instability.

Although not statistically significant, infants who developed post‐closure cardiorespiratory instability had higher E_ES_ both prior to and after intervention (Table 3, Table S3). There was a trend toward a higher E_ES_ after the procedure which may relate to anesthesia/medications or by inherent issues related to methodology. The pre‐procedural TnECHOs were done with alert infants while the post‐procedural scans were done while the infants were still sedated and/or muscle relaxed immediately after arrival from the catheterization laboratory. Irrespective of these limitations, our findings align with a previous study that described prolonged isovolumic relaxation time as a predictor for respiratory instability following PDA ligation in a targeted milrinone prophylaxis‐treated population (Ting et al., 2016). Isovolumic relaxation time is a marker of LV volume loading in the presence of a PDA which is also influenced by the inherent LV diastolic properties (Schmitz et al., 2004). Ventricular stiffening is associated with higher changes in pressure even in small loading perturbations, albeit at the expense of a larger changes in stroke work (Borlaug & Kass, 2008). Our study supports this finding given that the patients who developed instability had a smaller net change in SV but a larger drop in GLS and GWE. This is a novel finding which adds to the previous literature on the susceptibility to post‐PDA closure cardiorespiratory instability. Our data suggest that intrinsic myocardium properties related to diastolic function and ventricular stiffening may provide a basis for higher risk of ventriculo‐arterial uncoupling, decreased ventricular efficiency with relative higher myocardial oxygen consumption and an increased vulnerability to changes in loading conditions.

Our study also includes a novel method to study myocardial energetics using advanced 2D‐STE imaging. We demonstrated that myocardial work indices were feasible in this population with adequate intra‐ and inter‐observer reliability. The reliability of GLS and GWI is mainly dependent on tracing and tracking of the different myocardial segments. Meanwhile, the slightly lower reliability of GCW, GWW, and GWE is likely due to the definition of valvular time events. Since constructive and wasted work assess muscle contractility and relaxation in relation to the timing of the cardiac cycle, slight variations in the definition of the valvular time points are more likely to influence inter‐observer reliability. Our data support that changes in loading conditions seen in post‐PDA closure decrease overall longitudinal strain and work index. Furthermore, the changes in GCW, GWW, and GWE align with our results of the VAC model, such that post‐PDA closure is associated with decreased myocardial work efficiency. These findings provide the basis for the use of myocardial work analysis in other neonatal populations and in different physiological states.

### 4.1 Limitations

There are some important caveats to our study, particularly related to the retrospective design and the methods used to estimate VAC. *First*, as previously discussed, in the absence of invasive measurements and validation studies, it is not possible to determine which type (invasive vs. non‐invasive), location (pre‐ vs post‐ductal), and formula (ESP_1_, ESP_2_, and ESP_3_) is more reflective of “true” ESP in preterm infants and in those with PDA shunts. Additionally, the reliability of noninvasive arterial elastance estimation when the LV is exposed to both the systemic and the pulmonary vascular bed is not known. *Second*, neither Simpson's biplane nor stroke volume estimated by pulse‐wave Doppler has been validated in the estimations of arterial and end‐systolic elastance. There are also no studies evaluating the normative data for myocardial work in preterm infants. *Third*, in the estimation of E_ES_ it is appropriate to neglect V_0_ in patients with near normal loading conditions (Chowdhury et al., 2016) and therefore simplify the formula E_ES_ = ESP/(ESV‐V_0_) into E_ES_ = ESP/ESV (Chantler et al., 2008; Chowdhury et al., 2016; Tanoue et al., 2009). However, in patients with PDA and increased LV preload, V_0_ is increased, and therefore neglecting V_0_ may underestimate E_ES_ and overestimate E_A_/E_ES_. *Fourth*, we included a population in which most patients received milrinone prophylaxis. It is possible that milrinone, as a phosphodiesterase inhibitor with inotropic, lusitropic, and vasodilator properties (Akita et al., 1994; LeJemtel et al., 1989; Silver et al., 1989), may mitigate the impact of increased LV afterload on LV diastolic performance and in the development of post‐closure cardiorespiratory instability. Therefore, it is not possible to fully appraise the relationship of VAC and myocardial work indices with the development of instability in an untreated population. The study was also not designed or sufficiently powered to find predictors of cardiorespiratory instability. *Fifth*, our study only includes the TnECHO performed 1‐h after PDA closure with no further longitudinal data. It is possible that sustained changes in loading conditions, as well as the impact of weaning off from anesthesia, may further enhance changes in BP, and hence ESP and VAC estimations. *Lastly*, we did not assess fluid status in the patients included in this cohort. There were significant differences in pre‐procedural preload and stroke volume between patients with and without cardiorespiratory instability which could be attributed to differential fluid management. We believe that the changes in preload are most likely related to shunt volume given the relative homogeneity in fluid practices at our institution.

## 5 CONCLUSION

Percutaneous PDA closure leads to changes in ventriculo‐arterial coupling and myocardium energetics. An increase in arterial elastance, and hence LV afterload, associated with decreases in preload leads to decreased ventricular efficiency post‐closure. Furthermore, higher end‐systolic elastance may be associated with increased risk for post‐closure cardiorespiratory instability. These data provide novel insights in the physiology of PDA closure. It also enhances the understanding of the vulnerability to changes in loading conditions and may serve as a guide to optimize care and provide targeted therapies. Further studies are needed to validate measures of VAC in preterm infants according to PDA status and investigate the role of myocardial work indices in different physiological states in the neonatal population.

## CONFLICT OF INTEREST

The authors have no conflict of interest to disclose.

## AUTHOR CONTRIBUTIONS

Adrianne R Bischoff participated in the conception, design, acquisition, and analysis of data, drafted the article, and approved the final version. Amy H Stanford participated in acquisition of data, review of the manuscript for important intellectual content, and approved the final version. Patrick J McNamara participated in the conception, design, review of the manuscript for important intellectual content, and approved the final version.

## CATEGORY OF STUDY

Clinical.

## CONSENT STATEMENT

Patient consent was not required.

## Supporting information



Fig S1

Table S1‐S3
