# Supplementary figures and images for: Short‐term ventriculo‐arterial coupling and myocardial work efficiency in preterm infants undergoing percutaneous patent ductus arteriosus closure
Source: Physiol Rep. 2021 Nov 21;9(22):e15108. doi: 10.14814/phy2.15108 (PMC8606853; doi:10.14814/phy2.15108)

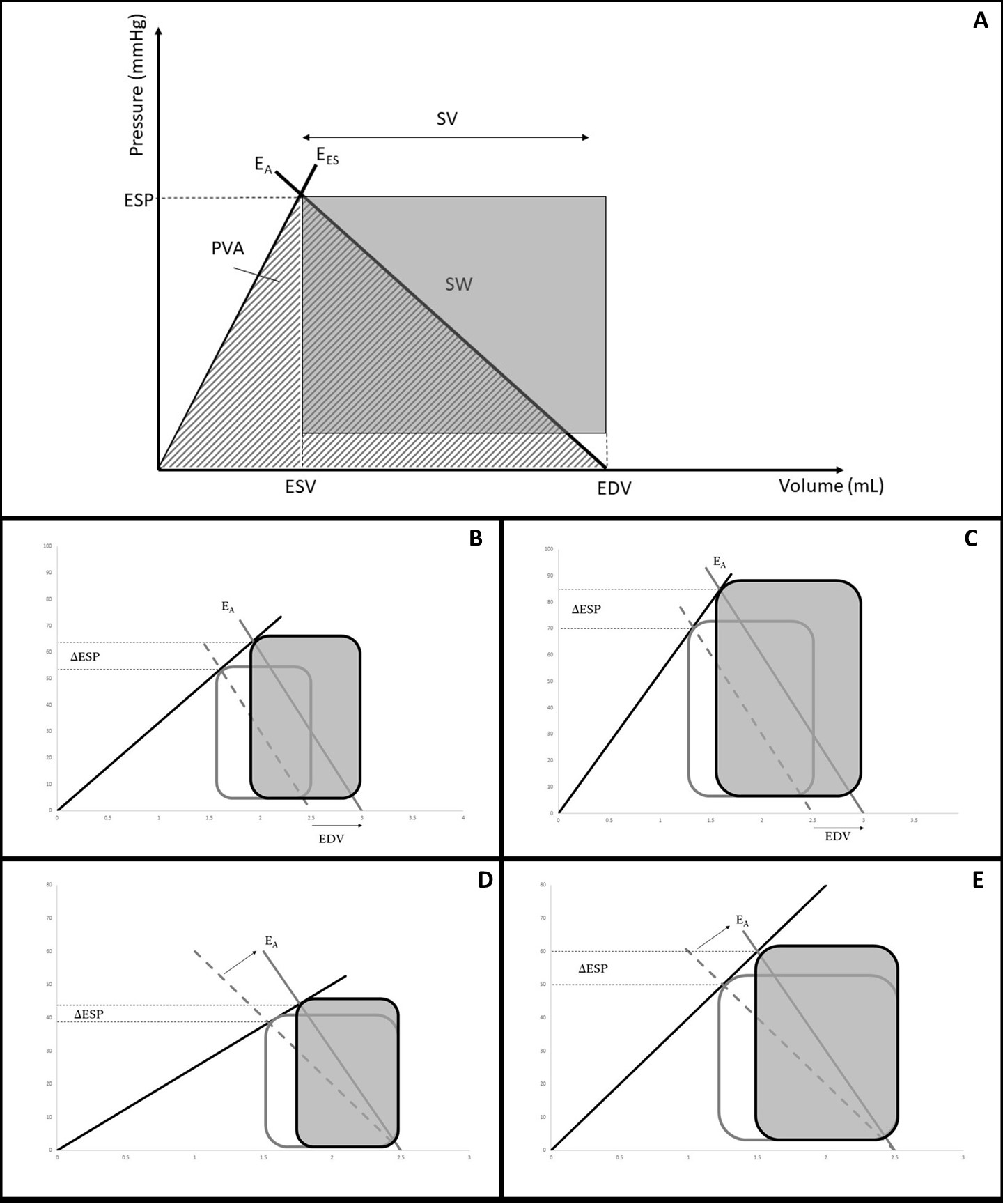

Supplement: Supplementary file 1 — Fig S1 [file PHY2-9-e15108-s002.jpg]
