# Supplementary material for: Short‐term ventriculo‐arterial coupling and myocardial work efficiency in preterm infants undergoing percutaneous patent ductus arteriosus closure
Source: Physiol Rep. 2021 Nov 21;9(22):e15108. doi: 10.14814/phy2.15108 (PMC8606853; doi:10.14814/phy2.15108)
Supplement: Supplementary file 2 — Table S1‐S3 [file PHY2-9-e15108-s001.docx]

**Supplemental Table 1:** Patent ductus arteriosus score, adapted from Rios *et al* (25).

| **Marker** | **0 points** | **1 point** | **2 points** |
| --- | --- | --- | --- |
| Mitral valve E-wave velocity, cm/sec | <45 | ≥45 and <80 | ≥80 |
| IVRT, msec | >45 | >30 and ≤45 | >45 |
| PV D-wave velocity, cm/sec | <30 | ≥30 and <50 | ≥50 |
| Left atrium/aorta ratio | <1.3 | ≥1.3 and <2.2 | ≥2.2 |
| LVO, mL/min/kg | <250 | ≥250 and <430 | ≥430 |
| Diastolic flow reversal in descending aorta and/or celiac/middle cerebral artery | no | ----- | yes |
| PDA diameter[mm]/weight[kg] | <1.5 | ≥1.5 and <3 | ≥3 |

IVRT: isovolumic relaxation time; PV: pulmonary vein; LVO: left ventricular output; PDA: patent ductus arteriosus

**Supplemental Table 2**: Pre- and post-procedural ventriculo-arterial coupling indices using alternative methods to calculate end-systolic pressure in preterm infants undergoing percutaneous closure of patent ductus arteriosus (n=35).

|  | **Pre-closure** | **1h Post-closure** | **p** |
| --- | --- | --- | --- |
| ESP_2_ (mmHg) | 57.3 ± 8.5 | 57.4 ± 10.3 | NS |
| ESP_3_ (mmHg) | 49.6 ± 9 | 50 ± 9.1 | NS |
| E_A3_ (mmHg/mL/kg) | 35.8 ± 10.6 | 59.1 ± 14.4 | <0.001 |
| E_A4_ (mmHg/mL/kg) | 32.2 [26.6, 43.2] | 55.3 [40.2, 68.4] | <0.001 |
| E_A5_ (mmHg/mL/kg) | 30.9 ± 9.4 | 48.9 ± 12.9 | <0.001 |
| E_A6_ (mmHg/mL/kg) | 27.7 [21.8, 37.9] | 48.9 [35.6, 59.5] | <0.001 |
| E_ES2_ (mmHg/mL/kg) | 65.1 [58.7, 103.4] | 85.9 [67.7, 115] | 0.004 |
| E_ES3_ (mmHg/mL/kg) | 56.2 [48.9, 90.6] | 76.7 [61.2, 96.1] | 0.009 |
| VAC_2_ | 0.5 ± 0.24 | 0.66 ± 0.32 | 0.011 |
| VE_2_ | 0.8 ± 0.06 | 0.76 ± 0.08 | 0.007 |

ESP: end-systolic pressure; E_A_: arterial elastance; E_ES_: end-systolic elastance; VAC: ventriculo-arterial coupling; VE: ventricular efficiency. Results presented in mean ± SD, median [IQR]. Paired t-test was used for normally distributed variables and Wilcoxon Signed Rank test for non-normally distributed variables with symmetry. Sign test was used for non-normally distributed variables without symmetry.

**Supplemental Table 3:** Ventriculo-arterial coupling parameters estimated by different formulas in patients with post-patent ductus arteriosus closure cardiorespiratory instability versus patients who remained clinically stable.

|  | **With cardiorespiratory instability (n=17)** | **Without cardiorespiratory instability (n=18)** | **p** |
| --- | --- | --- | --- |
| **Pre-procedure TnECHO** | | | |
| Stroke volume by LVO (mL/kg) | 1.73 ± 0.34 | 1.92 ± 0.57 | NS |
| ESP_2_ (mmHg) | 58 ± 7.4 | 56.7 ± 9.5 | NS |
| ESP_3_ (mmHg) | 50.8 ± 7.1 | 48.5 ± 10.5 | NS |
| E_A2_ (mmHg/mL/kg) | 39.7 ± 12.8 | 37.2 ± 12.7 | NS |
| E_A3_ (mmHg/mL/kg) | 36.8 [33.9, 39.6] | 29.8 [23.4, 47.9] | 0.067 |
| E_A4_ (mmHg/mL/kg) | 36.4 [29.2, 43.8] | 30.9 [25.7, 40.8] | NS |
| E_A5_ (mmHg/mL/kg) | 33.2 [29.1, 34.9] | 26.1 [19, 41] | 0.089 |
| E_A6_ (mmHg/mL/kg) | 31.9 [24.7, 39.5] | 26.9 [21.5, 36.5] | NS |
| E_ES2_ (mmHg/mL/kg) | 74.4 [63.6, 109.2] | 60.2 [54.3, 90.8] | 0.057 |
| E_ES3_ (mmHg/mL/kg) | 64.9 [54.7, 96.5] | 53.1 [43, 73.5] | NS |
| VAC_2_ | 0.46 [0.28, 0.67] | 0.45 [0.39, 0.58] | NS |
| VE_2_ | 0.81 [0.75, 0.87] | 0.81 [0.77, 0.84] | NS |
| **Post-procedure TnECHO** | | | |
| Stroke volume by LVO (mL/kg) | 1.07 ± 0.28 | 1.03 ± 0.31 | NS |
| ESP_2_ (mmHg) | 58.2 ± 7.9 | 56.6 ± 12.3 | 0.084 |
| ESP_3_ (mmHg) | 50.5 ± 7.1 | 49.6 ± 10.9 | 0.083 |
| E_A2_ (mmHg/mL/kg) | 59.9 ± 17.5 | 60.5 ± 21.5 | NS |
| E_A3_ (mmHg/mL/kg) | 57.5 [49.6, 71.9] | 46.4 [42.6, 68.2] | 0.029 |
| E_A4_ (mmHg/mL/kg) | 53.1 [39.7, 70.5] | 57.7 [40.2, 65.1] | NS |
| E_A5_ (mmHg/mL/kg) | 51.1 [43.5, 62.2] | 40.6 [36.6, 59] | 0.062 |
| E_A6_ (mmHg/mL/kg) | 48.9 [34.8, 59.7] | 49.3 [35.4, 59.5] | NS |
| E_ES2_ (mmHg/mL/kg) | 104.8 [83.6, 116.6] | 81.2 [54.7, 104] | 0.053 |
| E_ES3_ (mmHg/mL/kg) | 90.5 [76, 98.8] | 69.4 [48.3, 91.9] | 0.035 |
| VAC_2_ | 0.52 [0.41, 0.71] | 0.67 [0.5, 0.89] | NS |
| VE_2_ | 0.79 [0.74, 0.83] | 0.75 [0.69, 0.8] | NS |

BP: blood pressure; ESP: end-systolic pressure; LVO: left ventricular output; E_A_: arterial elastance; E_ES_: end-systolic elastance; VAC: ventriculo-arterial coupling; ESV: end-systolic volume; SV: stroke volume; VE: ventricular efficiency; GLS: global longitudinal strain; GWI: global myocardial work index; GCW: global constructive work; GWW: global wasted work; GWE: global work efficiency. Results presented in mean ± SD, median [IQR]. Parametric (Student t-test) and non-parametric tests (Mann-Whitney) were used as appropriate for continuous variables.
